# Editorial: Management of Females with Congenital Adrenal Hyperplasia

**DOI:** 10.3389/fped.2017.00282

**Published:** 2017-12-22

**Authors:** Ricardo González, Barbara Magda Ludwikowski

**Affiliations:** ^1^Pediatric Surgery and Urology, Kinder- und Jugendkrankenhaus AUF DER BULT, Hanover, Germany; ^2^Charité Universitätsmedizin Berlin, Berlin, Germany

**Keywords:** congenital adrenal hyperplasia, feminizing genitoplasty, vaginoplasty, clitoroplasty, disorders or differences of sex development

**Editorial on the Research Topic**

**Management of Females with Congenital Adrenal Hyperplasia**

The research topic “Management of females with congenital adrenal hyperplasia” attracted five manuscripts, four of which were accepted. The first to be discussed deals with the phenotypic expression of the mutation, two manuscripts deal with surgical techniques, and one with the controversial topic of the spectrum of disorders of sexual differentiation.

In the article entitled “Two Siblings with the Same Severe Form of 21-Hydroxylase Deficiency but Different Growth and Menstrual Cycle Patterns,” Lang-Muritano et al. from Zurich describe the cases of two sisters with an identical mutation followed from birth to adulthood that expressed quite different phenotypes despite excellent compliance with the prescribed hormonal replacement. Although the authors conclude that the differences were related to constitutional factors rather than to underlying congenital adrenal hyperplasia (CAH) mutation, the fact that the second sibling had a prenatal diagnosis and received *in utero* treatment, which successfully prevented virilization, cannot be ignored as a potential factor to explain differences in stature and menstrual cycles. Nevertheless, case reports of patients so carefully observed and followed till adulthood add valuable information to the understanding of any congenital anomaly.

K’efer and Rink’s article from Indianapolis on “Treatment of the Enlarged Clitoris” give an excellent overview of the anatomy of the clitoris and the techniques available to reduce the size of a hypertrophied organ. Clitoral reduction may be undertaken early, usually in response to parental concerns about the appearance of the external genitals of their daughter or later in life at the request of the affected individual. In any case, a thorough knowledge of the neural and vascular anatomy of the organ is essential to minimize the occurrence of the two worst complications of clitoral reduction namely atrophy and lack of sensitivity. Their review should provide essential knowledge to those called upon to undertake these procedures. We have become much more conservative in indicating this procedure, and in infants, we only perform it after the requesting caregivers have been extensively counseled and if the clitormegaly is so marked that it may clearly present a cosmetic problem for the child under usual circumstances. The article clearly describes various available techniques and the authors’ preference for a technique that preserves the tunica albuginea minimizing the risk of injury to the dorsal nerves. We concur completely with their opinion and the conclusions of the article.

Sircili et al. from Sao Paolo report their experience with the surgical management of 20 females with CAH who had undergone vaginoplasty early in childhood and presented with complications including recurrent urinary tract infection (UTI) in 9, dyspareunia in 6, and hematocolpos in 3 (2 associated with sepsis). The anatomical problems were persistence of UGS with stenosis in 17 patients and vaginal introitus stenosis in 3 patients (Sircili et al.).

The initial operation consisted of a Y-V flap in 60 patients 3 of whom presented later in life with complications. The remaining 17 patients had been operated elsewhere. At the time of the surgical revision, patients were in average 15 years old or about 13 years after the initial operation. Patients were followed up for a mean of 5 years after the revision. Success was achieved with 1 procedure in 15 women (75%), and the others required a second procedure. Vaginal dilatations were done after the revision in most patients.

In all, only 5% of the children operated initially at the author’s institution required revision, a considerably better outcome than reported by others (1, 2). We reserve Y-V flap vaginoplasty only for the least severe cases to avoid having an intravaginal urethral meatus, which could be responsible for UTIs after initiation of sexual intercourse. To avoid this complication, we prefer the *en bloc* mobilization of the UG sinus. It remains to be seen if this approach will yield a similar low incidence of vaginal stenosis. Nevertheless, it is encouraging to know that second surgery after puberty can yield good results when the operation was done in early childhood.

In the fourth paper in this collection (González and Ludwikowski), we postulate that given the controversies surrounding gender assignment and management of patients with conditions grouped under the umbrella term of DSD (disorders or differences of sex development), CAH in females just as hypospadias in boys should be excluded from this grouping. Not to repeat ourselves, we quote from the article: “Why should female gender assignment and early surgical correction in females with CAH be questioned and not male gender assignment and early correction in males with primary hypospadias? Consider the similarities of the two diseases: in both, the chromosomal sex is unequivocal, both can be successfully corrected with surgery leading to potentially normal sexual and reproductive functions, and in both gender, dysphoria is rare. Furthermore, reconstructive procedures for both conditions have less than perfect results. The complication rate and need for re-operations for fistulas persistent curvature and imperfect cosmetic appearance in cases of proximal hypospadias are comparable to the need for reoperation at puberty for introital stenosis with old operations in CAH.”

Excluding females with CAH from the DSD group would greatly simplify decision-making for both families and health care providers.

## Author Contributions

Both authors participated equally in the conception and writing of this editorial.

## Conflict of Interest Statement

The authors declare that the research was conducted in the absence of any commercial or financial relationships that could be construed as a potential conflict of interest.
